# Supplementary material for: Improved detection of differentially represented DNA barcodes for high‐throughput clonal phenomics
Source: Mol Syst Biol. 2020 Mar 18;16(3):e9195. doi: 10.15252/msb.20199195 (PMC7080434; doi:10.15252/msb.20199195)
Supplement: Supplementary file 1 — Appendix [file MSB-16-e9195-s001.pdf]

# Appendix material for "Improved detection of differentially represented DNA barcodes for high-throughput clonal phenomics"

Yevhen Akimov[1], Daria Bulanova[1,2], Sanna Timmonen[1], Krister Wennerberg[1,2], Tero Aittokallio[1,34,5*]

[1] Institute for Molecular Medicine Finland (FIMM), University of Helsinki, Tukholmankatu 8, Helsinki, 00290, Finland

[2] Biotech Research and Innovation Centre, University of Copenhagen, Ole Maaløes Vej 5, Copenhagen N, 2200, Denmark

[3] Department of Mathematics and Statistics, Quantum Building, University of Turku, 20014 Turku, Finland

[4] Department of Cancer Genetics, Institute for Cancer Research, Oslo University Hospital, N-0310 Oslo, Norway

[5] Oslo Centre for Biostatistics and Epidemiology (OCBE), Faculty of Medicine, University of Oslo, N-0317 Oslo, Norway

* Correspondence to Tero Aittokallio (tero.aittokallio@helsinki.fi)

Table of contents

- Appendix Table S1
- Appendix Table S2
- Appendix Table S3
- Appendix Table S4
- Appendix Table S5
- Appendix Figure S1
- Appendix Figure S2
- Appendix Figure S3
- Appendix Figure S4
- Appendix Figure S5
- Appendix Figure S6
- Appendix Figure S7
- Appendix Figure S8
- Appendix Figure S9
- Appendix Figure S10
- Appendix Figure S11
- Appendix Figure S12
- Appendix Figure S13

Appendix Table S1. Details of the benchmark dataset generated using DNA-barcoded cell pools.

| Cell line | Sample name as used in the main text | Number of sampled cells | Number of cells subsampled from the AB mix | Number of cells added from pool A | Sample name as used in read count tables (Dataset EV1 and EV2) | Number of replicas |
|---|---|---|---|---|---|---|
| OVCAR5 | Pool A | 1E+06 | - | - | P1 | 1 |
| OVCAR5 | Pool B | 1E+06 | - | - | P2 | 1 |
| OVCAR5 | Null-660 | - | 6,60E+05 | - | null_660.1; null_660.2 | 2 |
| OVCAR5 | Null-330 | - | 3,30E+05 | - | null_330.1; null_330.2 | 2 |
| OVCAR5 | Null-160 | - | 1,60E+05 | - | null_160.1; null_160.2 | 2 |
| OVCAR5 | Null-80 | - | 8,00E+04 | - | null_80.1; null_80.2 | 2 |
| OVCAR5 | Null-40 | - | 4,00E+04 | - | null_40.1; null_40.2 | 2 |
| OVCAR5 | Null-20 | - | 2,00E+04 | - | null_20.1; null_20.2 | 2 |
| OVCAR5 | name not used | - | 1,60E+05 | 5,60E+04 | m_null_160.p35.1; m_null_160.p35.2 | 2 |
| OVCAR5 | name not used | - | 1,60E+05 | 4,32E+04 | m_null_160.p27.1; m_null_160.p27.2 | 2 |
| OVCAR5 | name not used | - | 1,60E+05 | 2,88E+04 | m_null_160.p18.1; m_null_160.p18.2 | 2 |
| OVCAR5 | name not used | - | 8,00E+04 | 2,80E+04 | m_null_80.p35.1; m_null_80.p35.2 | 2 |
| OVCAR5 | name not used | - | 8,00E+04 | 2,16E+04 | m_null_80.p27.1; m_null_80.p27.2 | 2 |
| OVCAR5 | name not used | - | 8,00E+04 | 1,44E+04 | m_null_80.p18.1; m_null_80.p18.2 | 2 |
| OVCAR5 | name not used | - | 4,00E+04 | 1,40E+04 | m_null_40.p35.1; m_null_40.p35.2 | 2 |
| OVCAR5 | name not used | - | 4,00E+04 | 1,08E+04 | m_null_40.p27.1; m_null_40.p27.2 | 2 |
| OVCAR5 | name not used | - | 4,00E+04 | 7,20E+03 | m_null_40.p18.1; m_null_40.p18.2 | 2 |
| OVCAR5 | name not used | - | 2,00E+04 | 7,00E+03 | m_null_20.p35.1; m_null_20.p35.2 | 2 |
| OVCAR5 | name not used | - | 2,00E+04 | 5,40E+03 | m_null_20.p27.1; m_null_20.p27.2 | 2 |
| OVCAR5 | name not used | - | 2,00E+04 | 3,60E+03 | m_null_20.p18.1; m_null_20.p18.2 | 2 |
| Mia-PaCa-2 | Pool#1 (mia) | 1E+06 | - | - | Pool1 | 1 |
| Mia-PaCa-2 | Pool#2 (mia) | 1E+06 | - | - | Pool2 | 1 |
| Mia-PaCa-2 | Null-40 (mia) | | 4,00E+04 | - | sampl40_1; sampl40_2; sampl40_3; | 3 |
| Mia-PaCa-2 | Null-10 (mia) | | 2,00E+04 | - | sampl10_1; sampl10_2; sampl10_3; | 3 |

- stands for “not applicable”

Appendix Table S2. Details of the benchmark carboplatin phenotyping dataset.

| Cell line | Assay | Collection time point (day) | Number of cells seeded (x$10^6$) | Sample name as used in read count table (Dataset EV4) | Number of replicas |
|---|---|---|---|---|---|
| OVCAR5 | Carboplatin treatment (7uM, 4 days); reference samples | 8 | 3 | X15cm_1, X15cm_2, X15cm_3, X15cm_4 | 4 |
| OVCAR5 | Carboplatin treatment (7uM, 4 days); subsamples | 8 | 1.3 | X10cm_1, X10cm_2 | 2 |
| OVCAR5 | Carboplatin treatment (7uM, 4 days); subsamples | 8 | 0.16 | X6wp_1, X6wp_2 | 2 |
| OVCAR5 | Carboplatin treatment (7uM, 4 days); subsamples | 8 | 0.067 | X12wp_1, X12wp_2 | 2 |
| OVCAR5 | Growth | 0 | 3 | growth_081119_1, growth_081119_2 | 2 |
| OVCAR5 | Growth; control | 4 | 3 | growth_121119_1, growth_121119_3, growth_121119_3, growth_121119_4 | 4 |
| OVCAR5 | Growth | 11 | 3 | growth_191119_1, growth_191119_2 | 2 |

Appendix Table S3. Details of the OVCAR5 clone phenotyping experimental dataset.

| Cell line | Assay | Collection time point (day) | Number of cells collected (x$10^6$) | Sample name as used in read count table (Dataset EV4) | Number of replicas |
|---|---|---|---|---|---|
| OVCAR5 | Growth | 0 | 3 | T0 | 1 |
| OVCAR5 | Growth | 5 | 3 | T5 | 1 |
| OVCAR5 | Growth | 8 | 3 | T8 | 1 |
| OVCAR5 | Growth | 11 | 3 | T11_1; T11_2; T11_3; T11_4 | 4 |
| OVCAR5 | Growth | 14 | 3 | T14_1; T14_2; T14_3 | 3 |
| OVCAR5 | Growth | 18 | 3 | T18_1; T18_2; T18_3 | 3 |
| OVCAR5 | Growth | 25 | 3 | T25_1; T25_2; T25_3 | 3 |
| OVCAR5 | Growth | 29 | 3 | T29_1; T29_2; T29_3 | 3 |
| OVCAR5 | Efflux (HIGH) | 8 | 0.2 | Efflux_pos_1; Efflux_pos_2 | 2 |
| OVCAR5 | Efflux (LOW) | 8 | 0.35 | Efflux_neg_1; Efflux_neg_2 | 2 |
| OVCAR5 | ALDH (HIGH) | 8 | 0.25 | ALDH_pos_1; ALDH_pos_2 | 2 |
| OVCAR5 | ALDH (LOW) | 8 | 0.6 | ALDH_neg_1; ALDH_neg_2 | 2 |
| OVCAR5 | Autophagy (HIGH) | 11 | 0.1 | Autophagy_pos_1; Autophagy_pos_2 | 2 |
| OVCAR5 | Autophagy (LOW) | 11 | 0.12 | Autophagy_neg_1; Autophagy_neg_2 | 2 |
| OVCAR5 | Attachment (HIGH) | 11 | 3 | Attach_pos_1; Attach_pos_2 | 2 |
| OVCAR5 | Attachment (LOW) | 11 | 3 | Attach_neg_1; Attach_neg_2 | 2 |
| OVCAR5 | KI67 (HIGH) | 11 | 0.15 | KI67_pos_1; KI67_pos_2 | 2 |
| OVCAR5 | KI67 (LOW) | 11 | 0.15 | KI67_neg_1; KI67_neg_2 | 2 |
| OVCAR5 | Carboplatin 7uM | 11 | 3 | Carb_11_1; Carb_11_2 | 2 |
| OVCAR5 | Carboplatin 7uM | 14 | 3 | Carb_14_1; Carb_14_2 | 2 |
| OVCAR5 | Carboplatin 7uM | 18 | 3 | Carb_18_1; Carb_18_2 | 2 |

Appendix Table S4. Golden gate protocol for DNA barcode cloning into B-GLI-Barcoding plasmid

| Golden gate protocol | | |
|---|---|---|
| Rapid Ligation Buffer (Thermo, cat. K1422) | 4 µl | |
| AarI (2U/ul) (Thermo, cat. ER1581) | 1 µl | |
| T4 DNA Ligase (5 U/µL) (Thermo, cat. EL0014) | 0.25 µl | |
| Barcode amplicon | 5 ng | |
| B-GLI-Barcoding | 100 ng | |
| Oligo ( provided with the AarI enzyme) | 0.4 µl | |
| ddH2O | up to 20 µl | |
| Golden gate cycling conditions | | |
| Temperature | Time | Cycles |
| 37C | 5 min | 1 |
| 37C<br>22C | 5 min<br>3 min | 50 |
| 37C | 10 min | 1 |
| 65C | 15 min | 1 |
| 4C | hold | 1 |

Appendix Table S5. **β** threshold estimation rules.

| | min(overlap)>0.25 | min(overlap)<0.25 |
|---|---|---|
| Descending sigmoid curve | Data follows NB model; beta set to 0 | Visual examination of the results is recommended; user-specified beta is used |
| Ascending sigmoid curve | Data follows NB model; beta set to 0 | max(overlap)<0.25<br>Data does not follow NB; user-specified beta is used<br>max(overlap)>0.25<br>beta is estimated as described in methods |

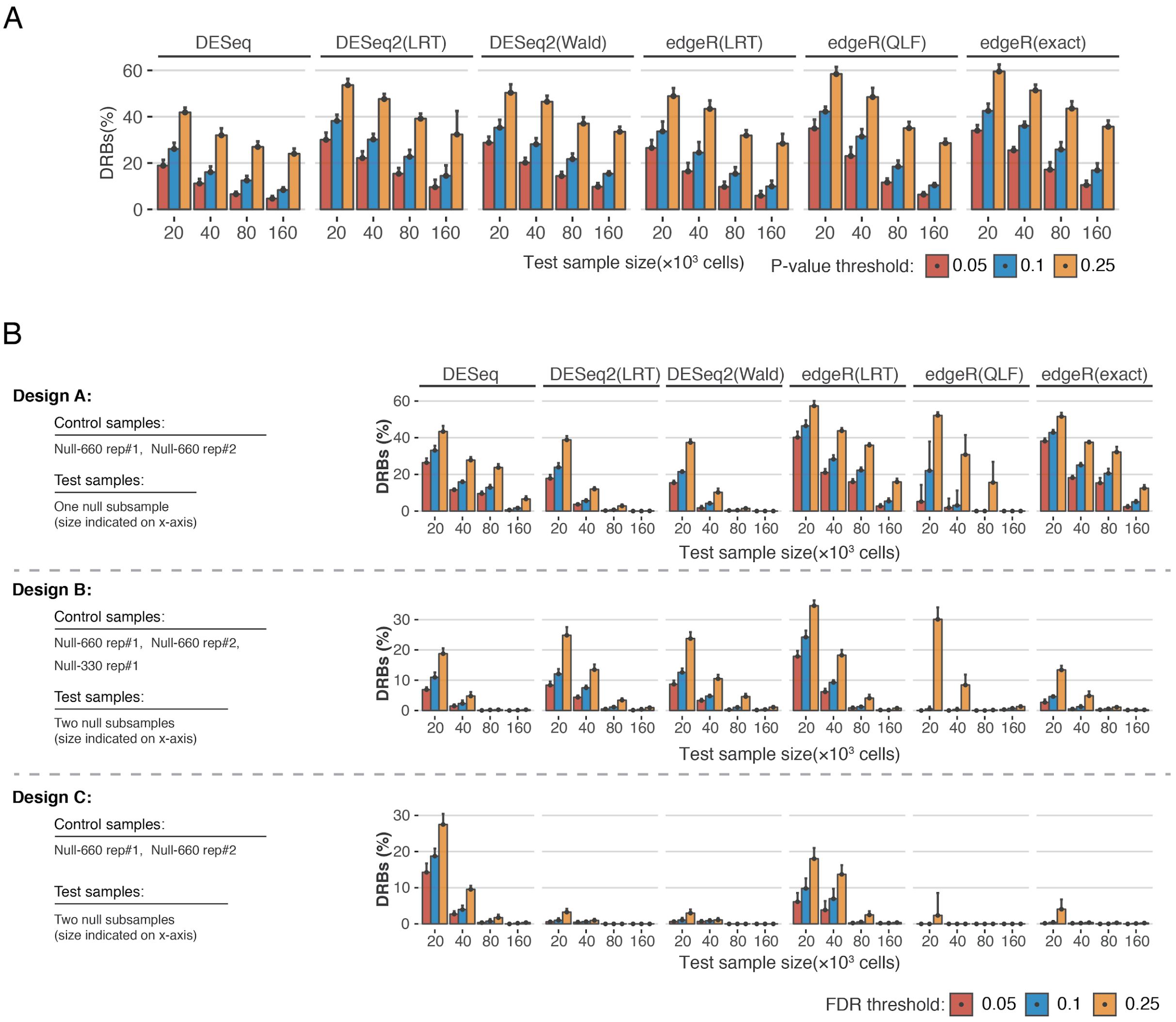


**Appendix Figure S1.**

**Comparison of the RNA-seq algorithms' performance on the null subsamples.**

A. The proportion of differentially represented barcodes (DRBs) identified in the null subsamples with various RNA-seq analysis algorithms using the same design as in Fig 2D. The bars represent the mean proportion of DRBs calculated over 3-fold bootstrap runs (mean of the 10 resamples with replacement) under indicated unadjusted p-value thresholds.

B. Bars represent the proportion of DRBs identified in the OVCAR5 null subsamples with different RNA-seq analysis algorithms. Samples used in control and treatment groups are indicated on the left. The bars represent the average percentage of barcodes called falsely as differentially represented by the indicated algorithms (10 resamplings with replacement), under different nominal false discovery rate (FDRs) thresholds.

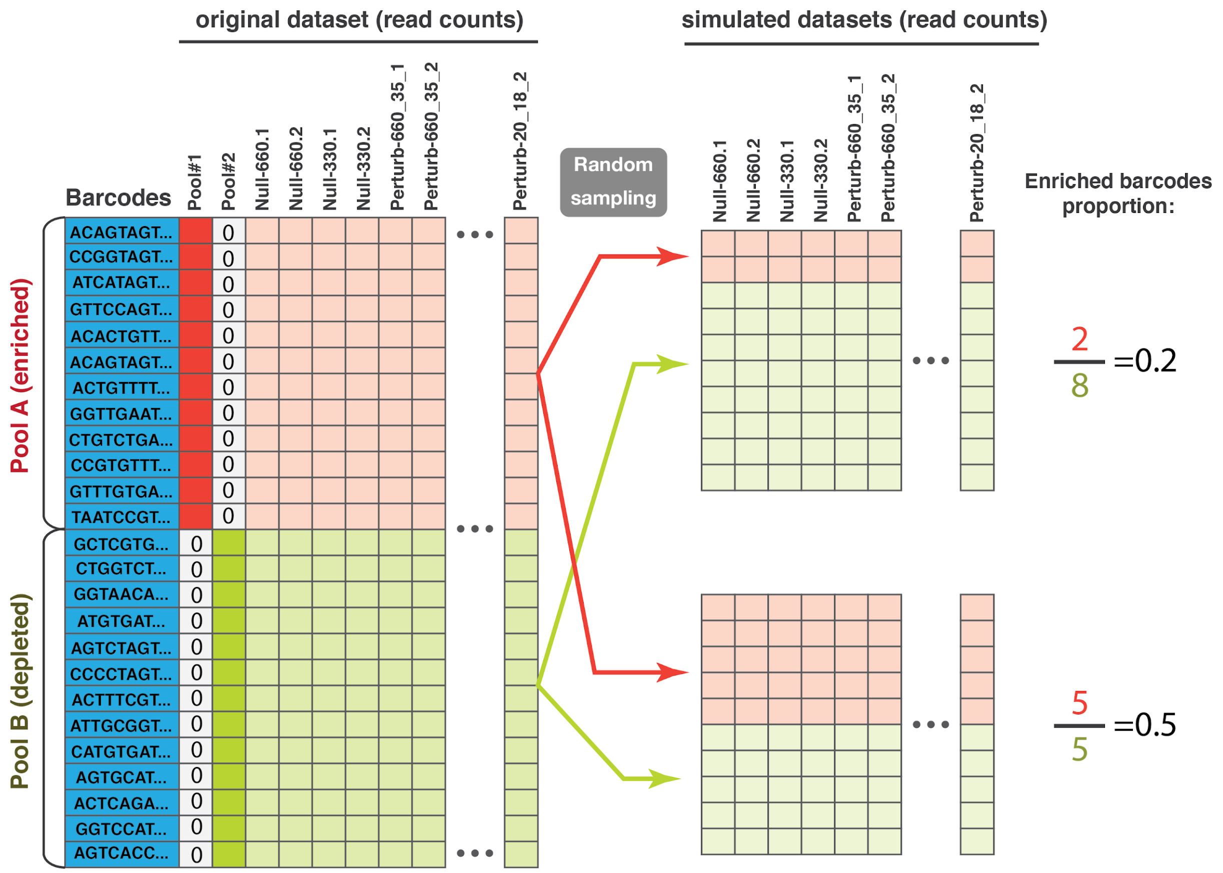


**Appendix Figure S2.**

**Modelling of barcode experimental results with varying enriched barcode proportions.**

The barcodes are assigned into enriched and depleted groups according to the ground-truth (defined by sequencing of the cell pools A and B). Next, the barcodes are sampled without replacement to the desired percentage of enriched. Note that after normalization the original perturbation degrees are subject to change, depending on the chosen enriched ratio.

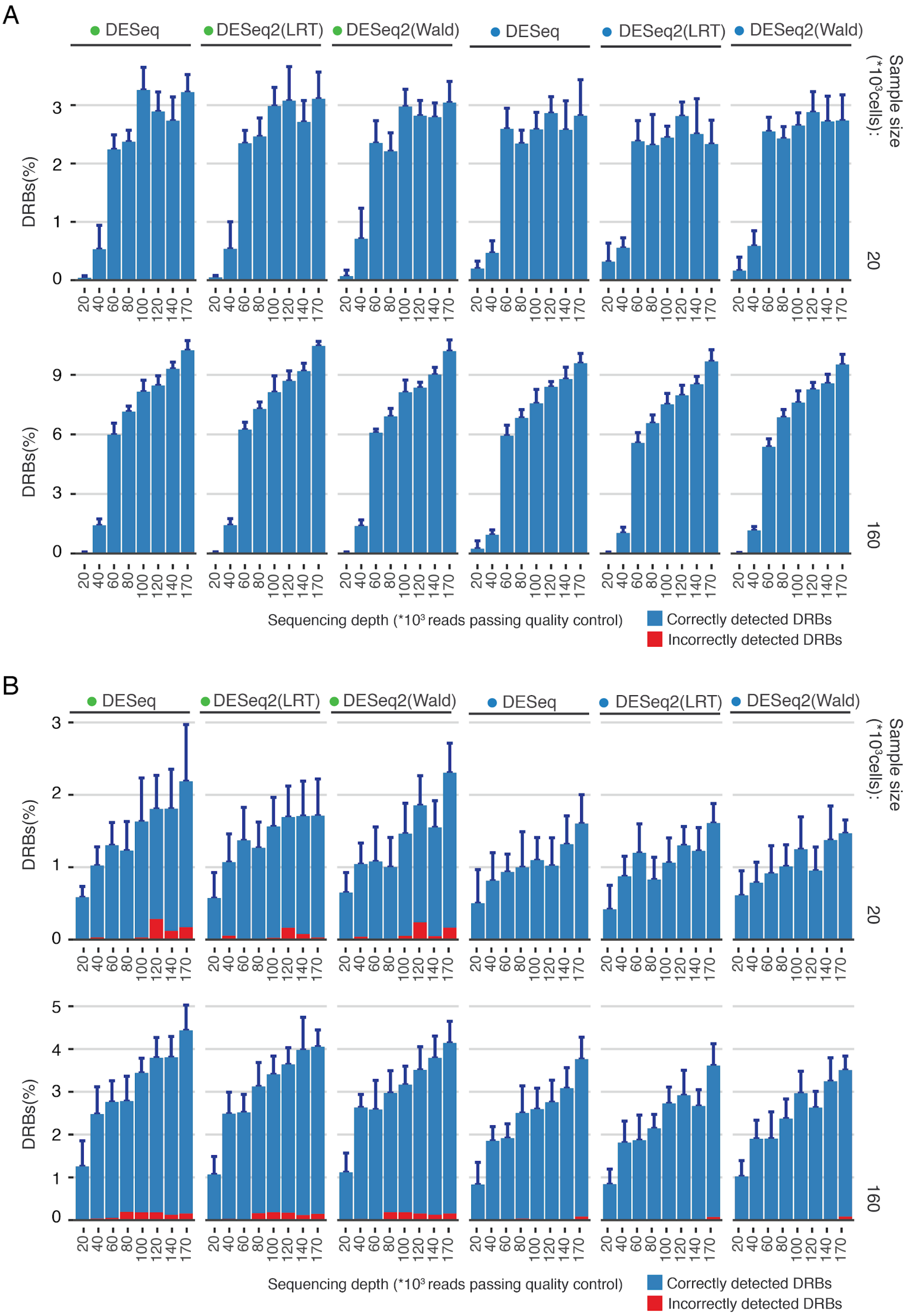


**Appendix Figure S3.**

**Effect of sequencing depth on performance of the modified versions of the DESeq2 and DESeq algorithms.**

A-B. Proportion of the DRBs detected by the algorithms for samples with perturbation degree of 35% and indicated size (left). Tests are performed for the samples with enriched proportions of 0.5 (A) and 0.15 (B).

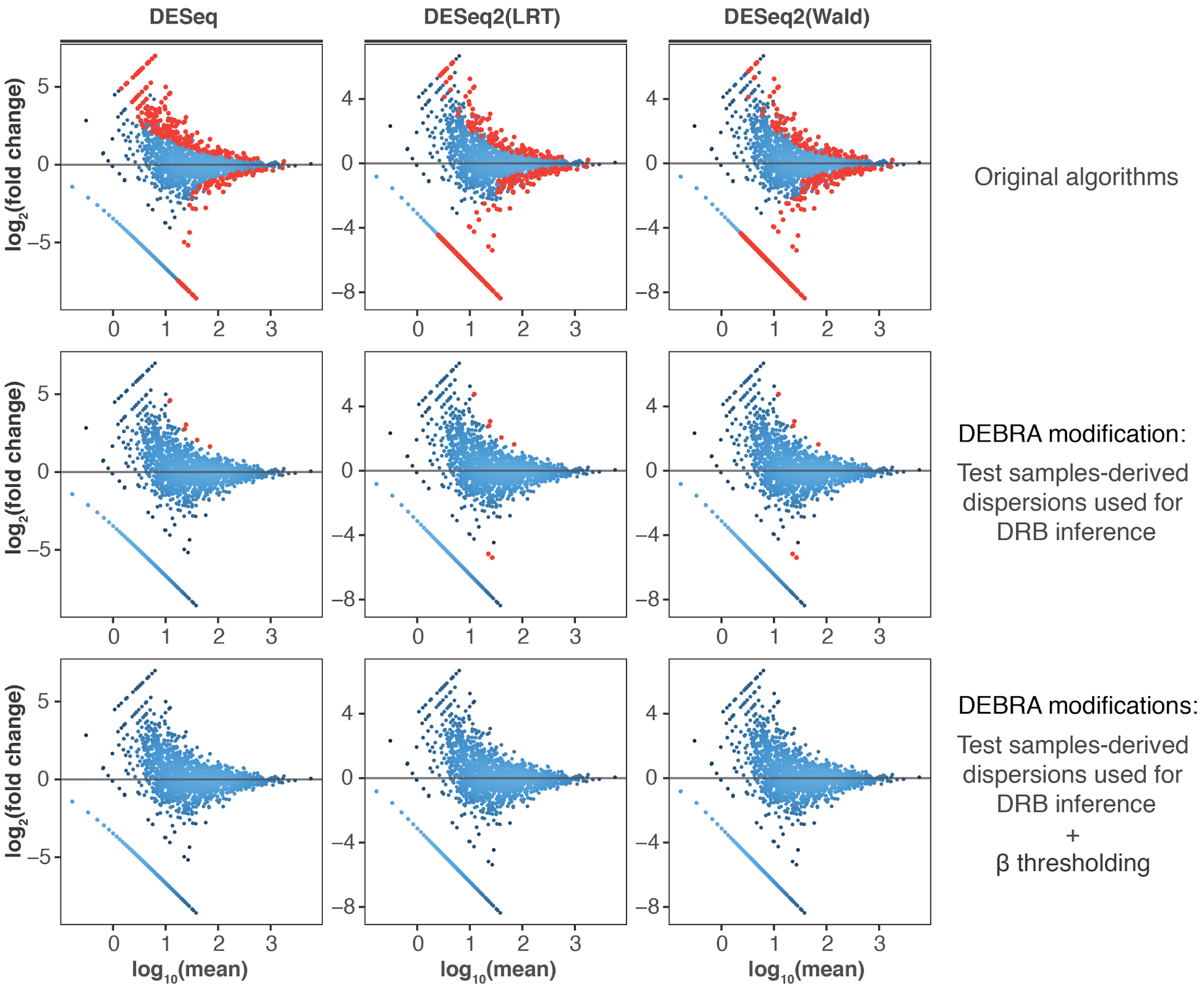


**Appendix Figure S4.**

**The effect of the modifications implemented in DEBRA on the false discovery rate.**

MA-plots for the DRB detection results of various versions of the algorithms (top) and their modifications (right) on the null-20 subsample (two replicas) tested against four null subsamples (two null-660 and two null-330). Barcodes with FDR>0.1 are marked in red.

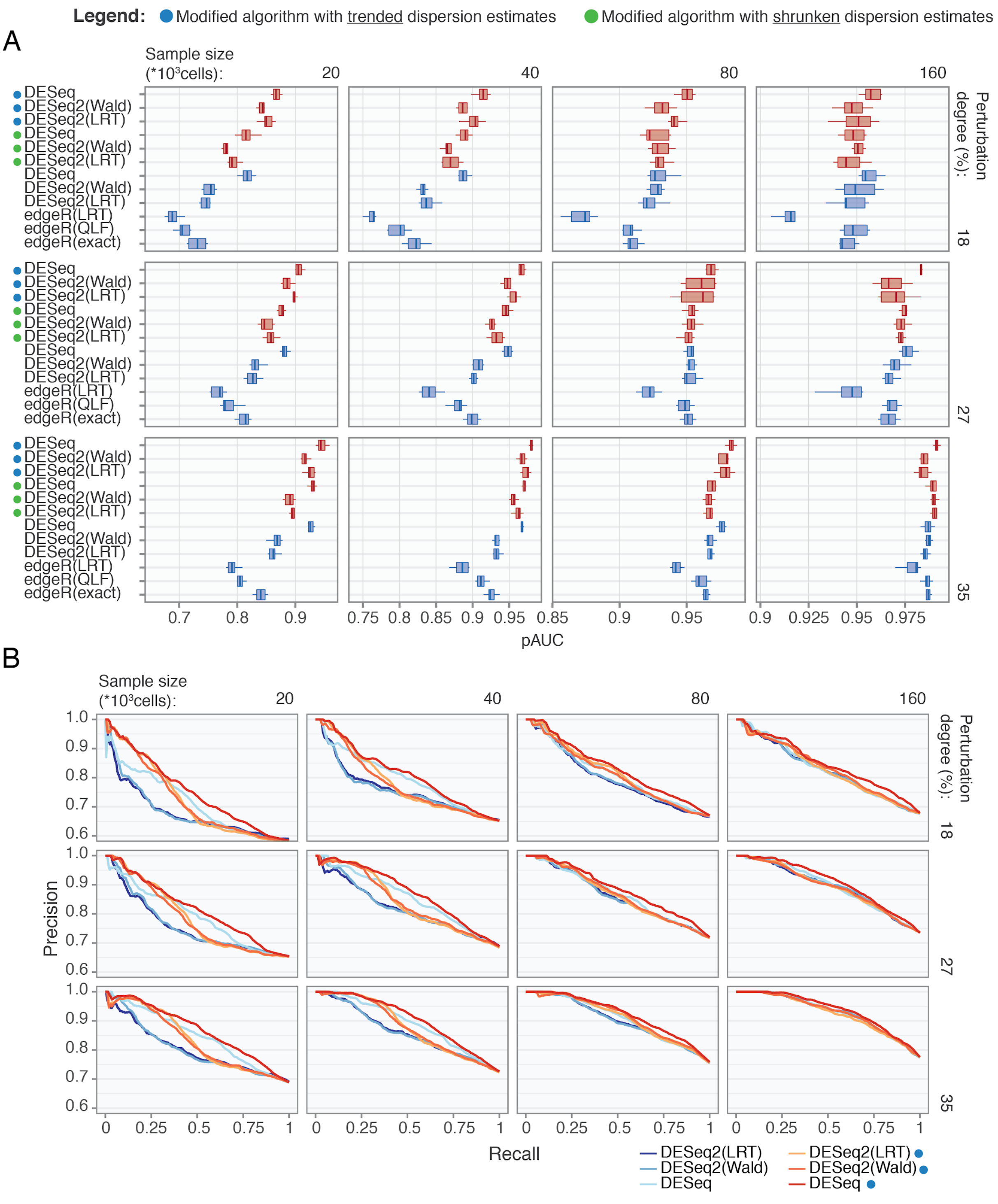


**Appendix Figure S5.**

**Comparison of the DRB scoring performance using precision-recall analysis.**

Circles left to the algorithms' names indicate the modified algorithms.

A. Standardized partial area under the precision-recall curve (pAUC) calculated within intervals of [0,1] for precision and [0, X] for recall metrics where X is the mean recall value at FDR=0.25 for a given sample across all tested algorithms. Shown are the pAUCs for perturbed subsamples of indicted size (top) and perturbation degree (right), with enriched barcodes proportion of 0.5.

B. Precision-recall curves for the indicated sample sizes (top) and perturbation degrees (right), with enriched barcodes proportion of 0.5. For clarity, the modified algorithms with shrunken dispersion estimates are not shown.

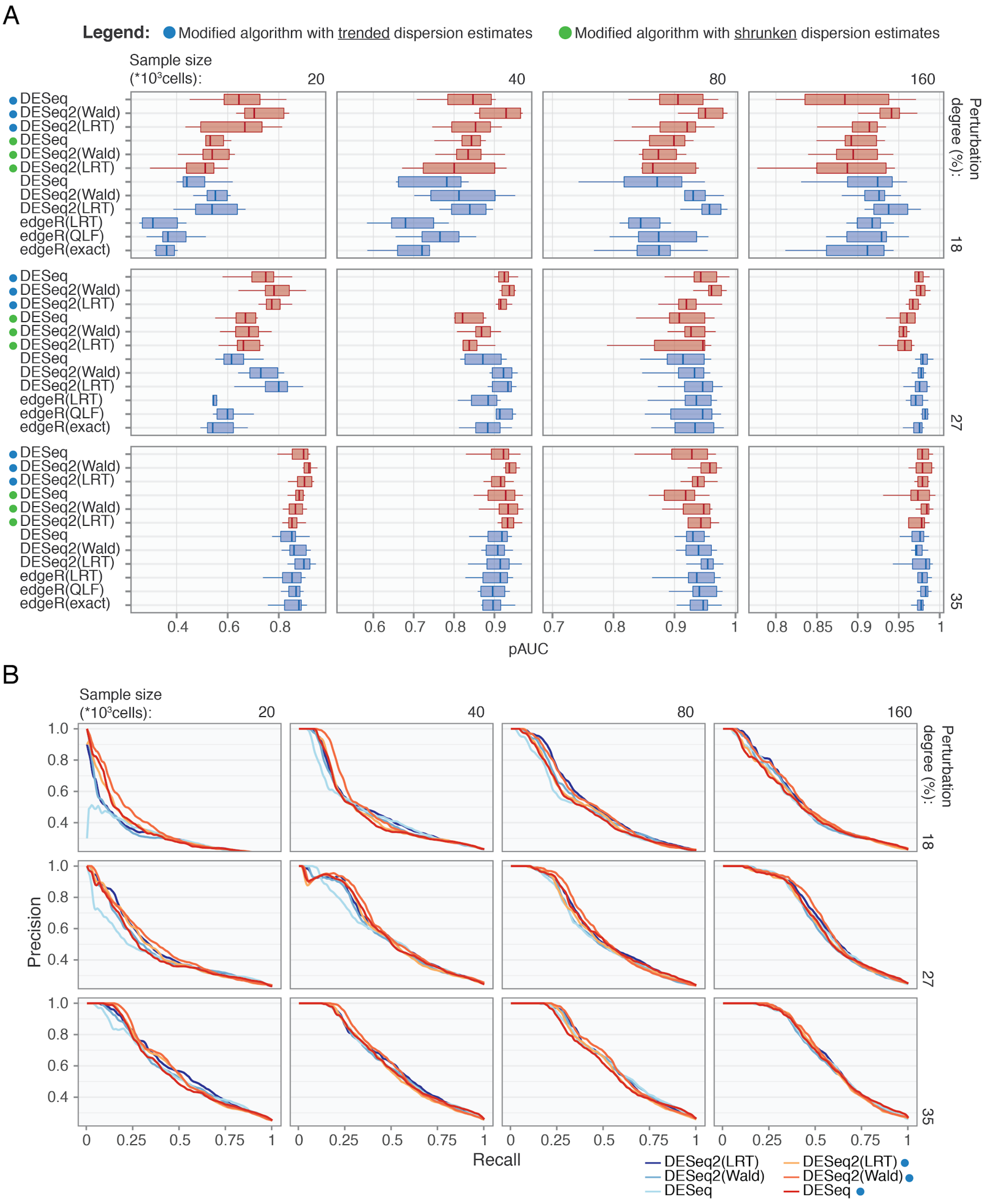


**Appendix Figure S6.**

**Comparison of the DRB scoring performance using precision-recall metrics.**

Circles left to the algorithms' names indicate the modified algorithms.

A. Standardized partial area under the precision-recall curve (pAUC) calculated within intervals of [0,1] for precision and [0, X] for recall metrics where X is the mean recall value at FDR=0.25 for a given sample across all tested algorithms. Shown are the pAUCs for perturbed subsamples of indicted size (top) and perturbation degree (right), with enriched to depleted barcodes ratio of 0.15.

B. Precision-recall curves for the indicated sample sizes (top) and perturbation degrees (right), with enriched to depleted barcodes ratio of 0.15. For clarity, the modified algorithms with shrunken dispersion estimates are not shown.

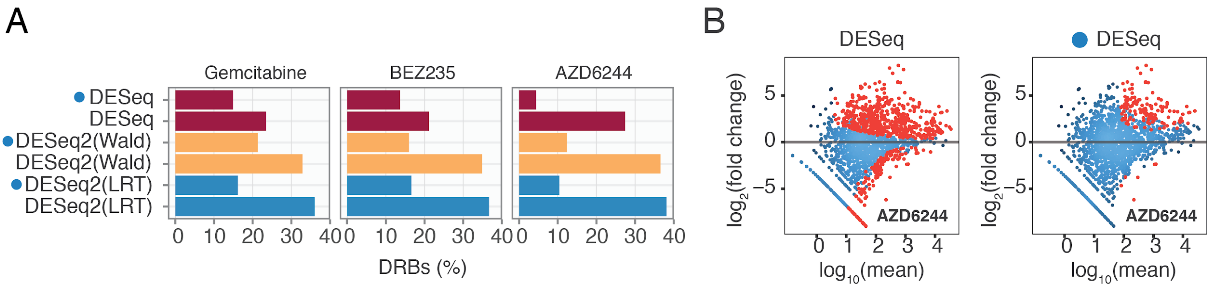


**Appendix Fig S7.**

**DEBRA performance on the Seth *et. al.* pancreatic PDX dataset.**

A. The proportion of significant DRBs with FDR < 0.1 in the Seth et al. pancreatic PDX dataset, as identified with the modified and original algorithms.

B. Log fold change vs log mean plots for the AZD6244 drug-treated samples tested against untreated control with the original DESeq and modified (trended) DESeq algorithms. Red dots mark barcodes with FDR < 0.1.

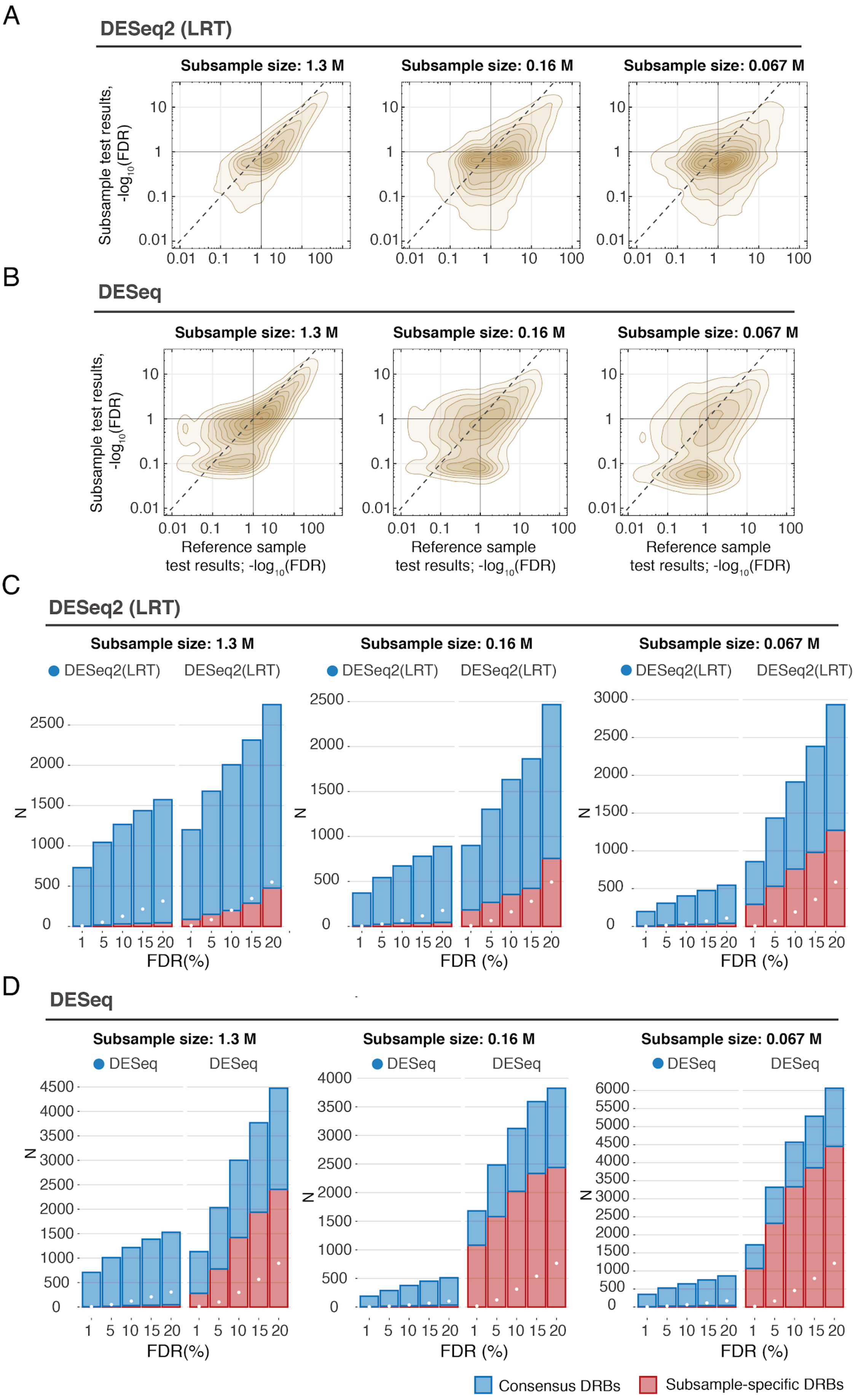
A
DESeq2 (LRT)
Subsample size: 1.3 M
Subsample size: 0.16 M
Subsample size: 0.067 M
Subsample test results, -log10(FDR)
B
DESeq
Reference sample test results; -log10(FDR)
C
DESeq2 (LRT)
DESeq2(LRT)
N
FDR(%)
D
DESeq
Consensus DRBs
Subsample-specific DRBs

**Appendix Fig S8. Comparison of algorithms' performance for carboplatin response phenotype.**

A-B. Density plots of FDR values estimated for reference sample (x-axis) plotted against corresponding FDR values of subsamples of different sizes (y-axis) estimated with (A) DESeq2(LRT) and (B) DESeq. The region where barcodes have FDR values lower than the set threshold of 0.1 for both reference and subsamples (consensus DRBs) are outlined with a blue square. Red square outlines the region where the barcodes are detected as significant only in subsample test (subsample-specific DRBs) with total number indicated in the top-left corner.

C-D. Number of DRBs with FDR lower than a threshold (x-axis) detected with (C) DESeq2(LRT) and (D) DESeq algorithms for subsamples of indicated sizes (top). Blue bars represent the number of barcodes detected as significant in both reference and subsample (consensus DRBs), red bars represent the number of subsample-only DRBs. White circles mark the percentage of barcodes corresponding to the nominal FDR level.

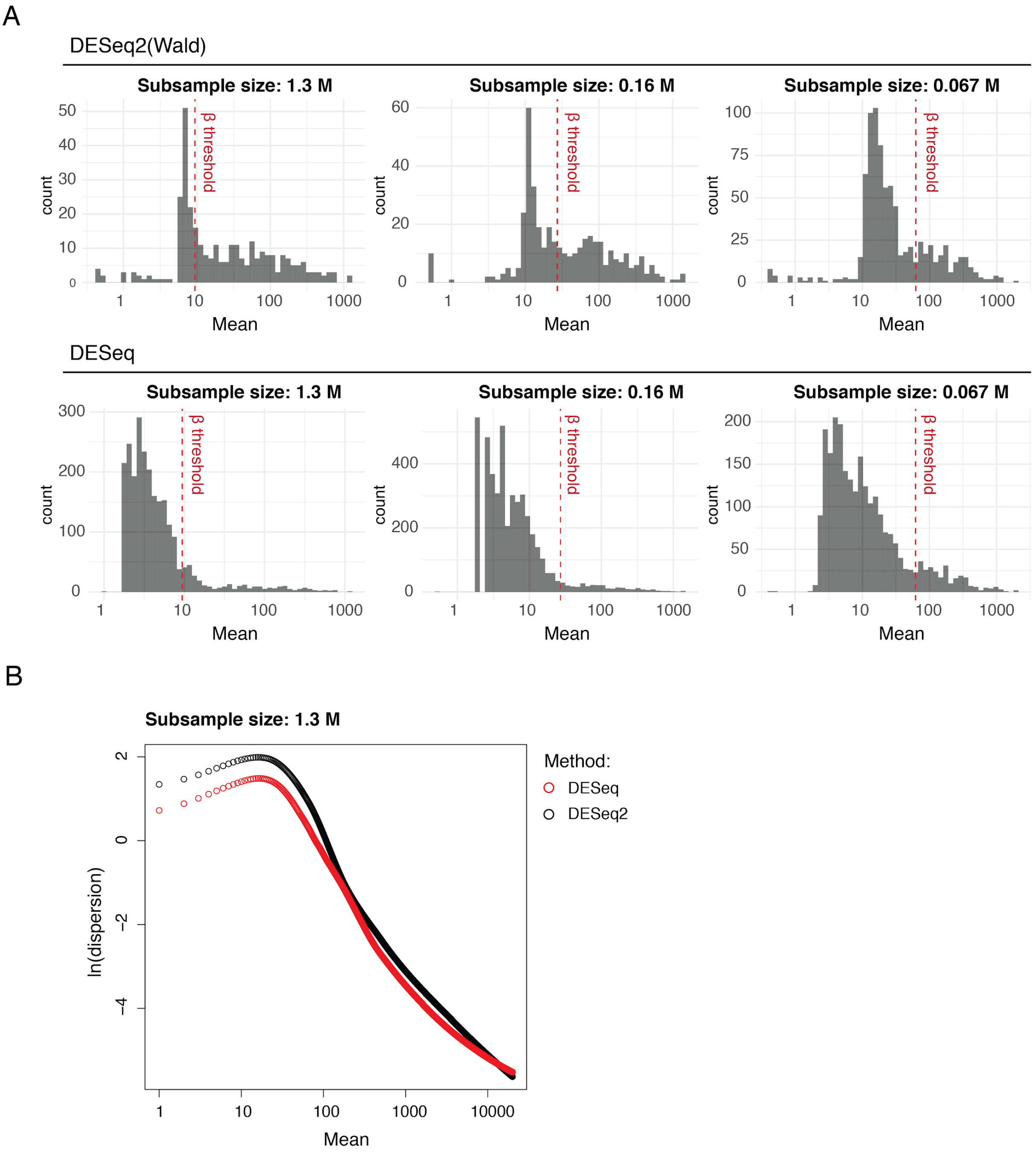


**Appendix Fig S9. Characteristics of the subsample-specific DRBs**

A. Histogram of the mean read counts for the DRBs detected in the subsamples but not in the reference samples by DESeq2(Wald) or DESeq. Dashed red line indicate the beta threshold.

B. Mean - dispersion relationship as fitted by DESeq or DESeq2 for subsample-specific DRBs. Dispersion fits were extracted using *object@dispersionFunction* for DESeq2 and with *object@fitInfo[["pooled"]][["dispFunc"]]* for DESeq.

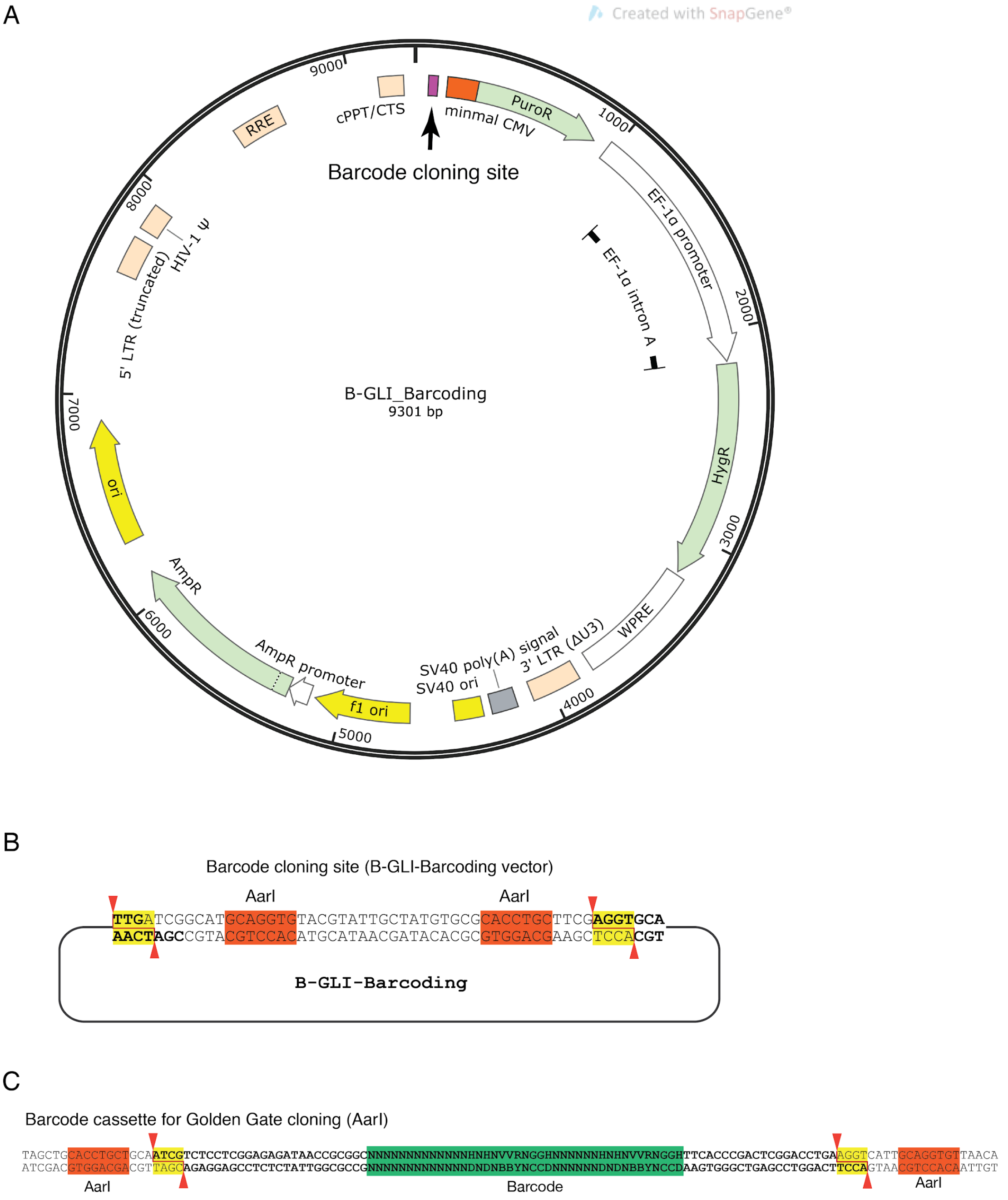


**Appendix Figure S10.**

**Lentiviral barcoding plasmid and cloning strategy outline**

A. B-GLI-Barcoding plasmid map; image generated using SnapGene software (from GSL Biotech; available at snapgene.com)
B. Cloning site of B-GLI-Barcoding plasmid. AarI cut sites are marked in yellow.
C. Amplified barcode cassette as used for barcode cloning into B-GLI-Barcoding vector.

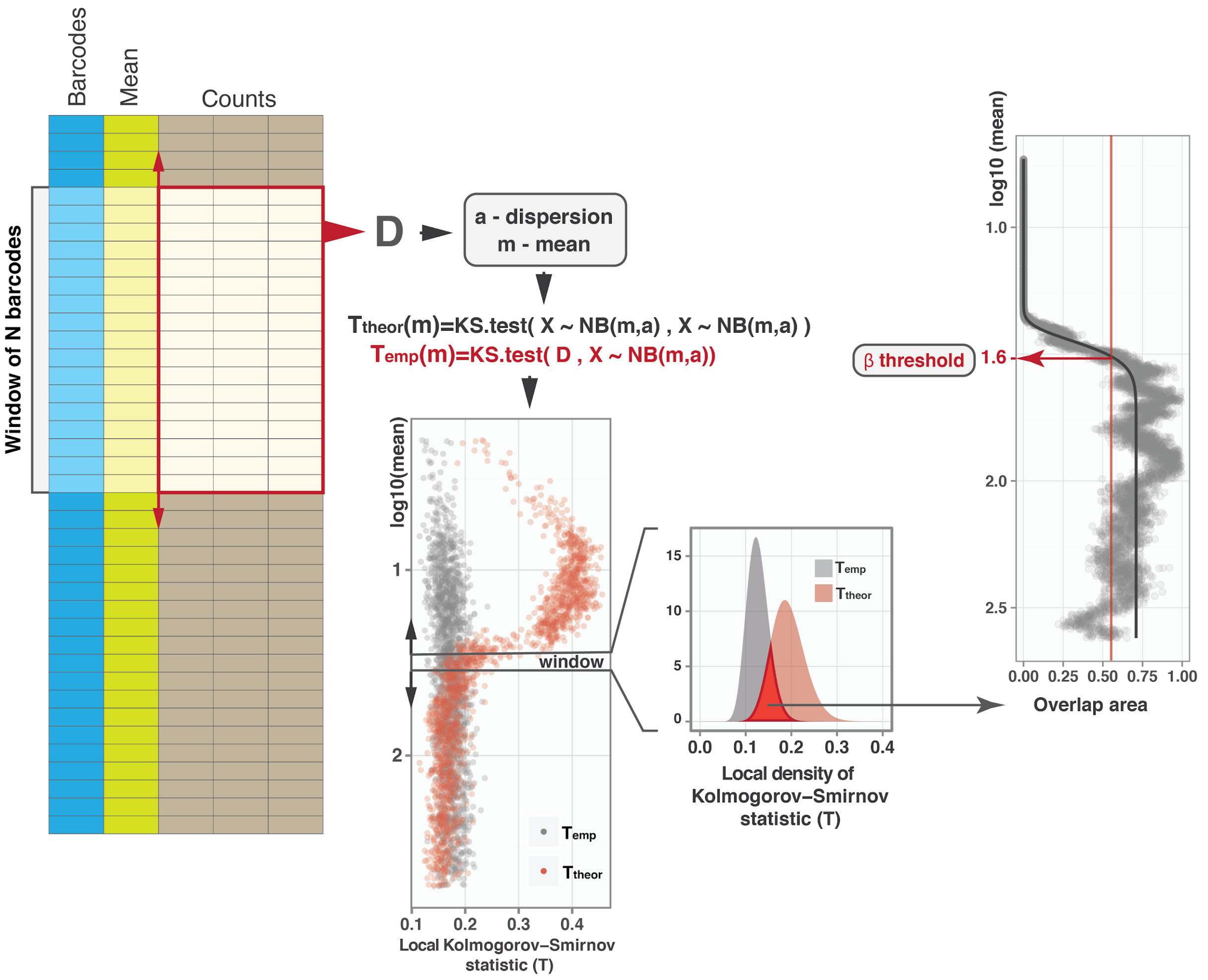


**Appendix Figure S11.**

**The steps of the β threshold estimation algorithm.**

The algorithm samples read count data ordered by the mean count value to obtain local estimates of mean and dispersion parameters of the negative binomial (NB) model. For each sampling window, theoretical and empirical Kolmogorov-Smirnov (KS) statistic values are calculated. Theoretical KS statistics is estimated on two sets of random negative binomial variables, whilst the empirical KS statistic is obtained by testing sampled data vs random negative binomial variables simulated using the previously estimated NB distribution parameters. After local estimation of these KS statistic values, the algorithm performs a local fitting of the empirical and theoretical KS statistic with a Gamma-distribution, and the overlap of local densities is fitted as a function of the mean read count using a 4-parameter sigmoid function. The **β** threshold is the value of the mean at which the fitted overlap takes the value of 0.8 of maximum.

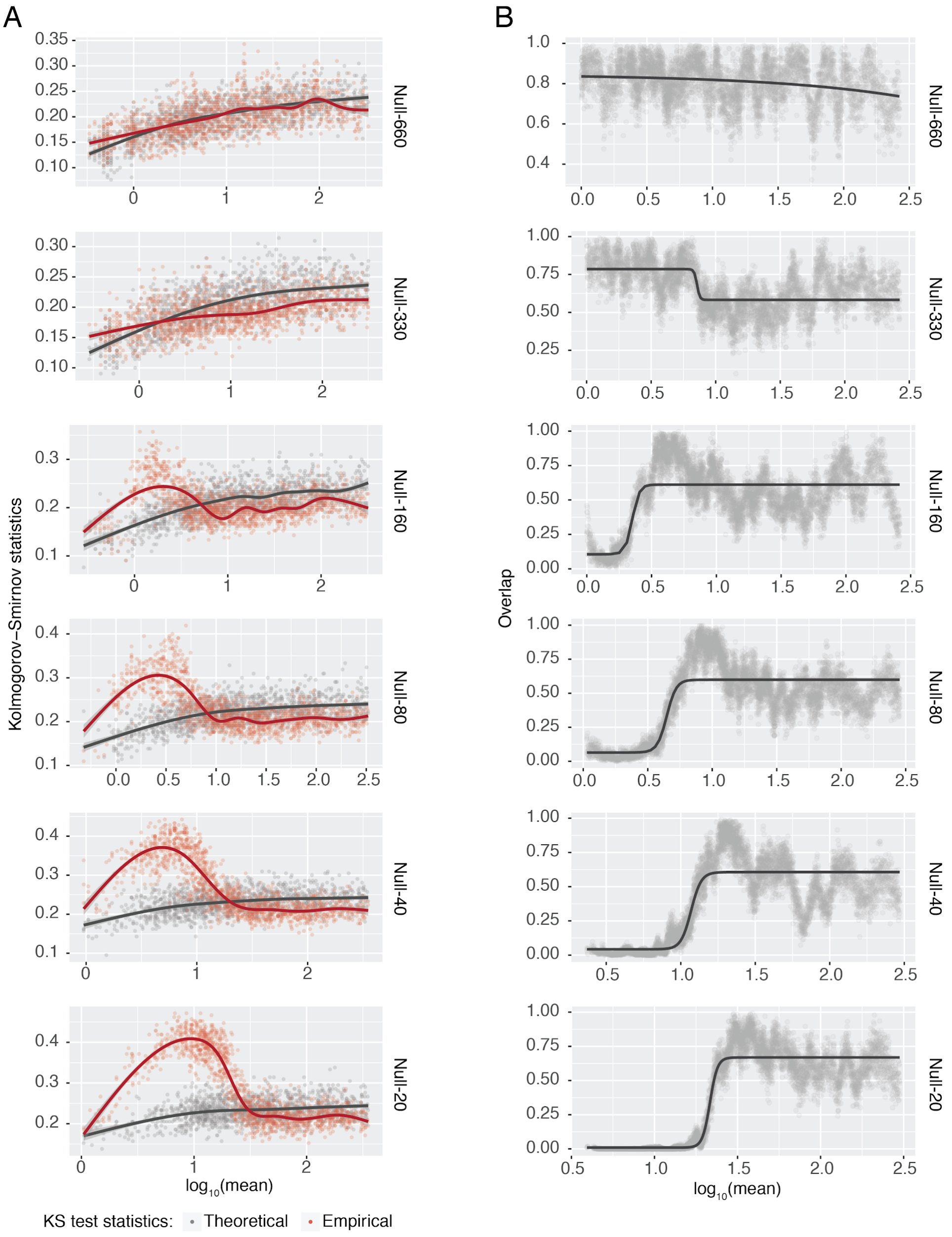


**Appendix Figure S12.**

**β threshold estimation algorithm applied to the null subsamples.**

A. Empirical and theoretical (see Methods) local two-sample Kolmogorov-Smirnov test statistics for the null subsamples of different sizes.

B. Estimation of the local overlap between Gamma-distributed empirical and theoretical Kolmogorov-Smirnov test statistics fitted with four parameters sigmoid function.

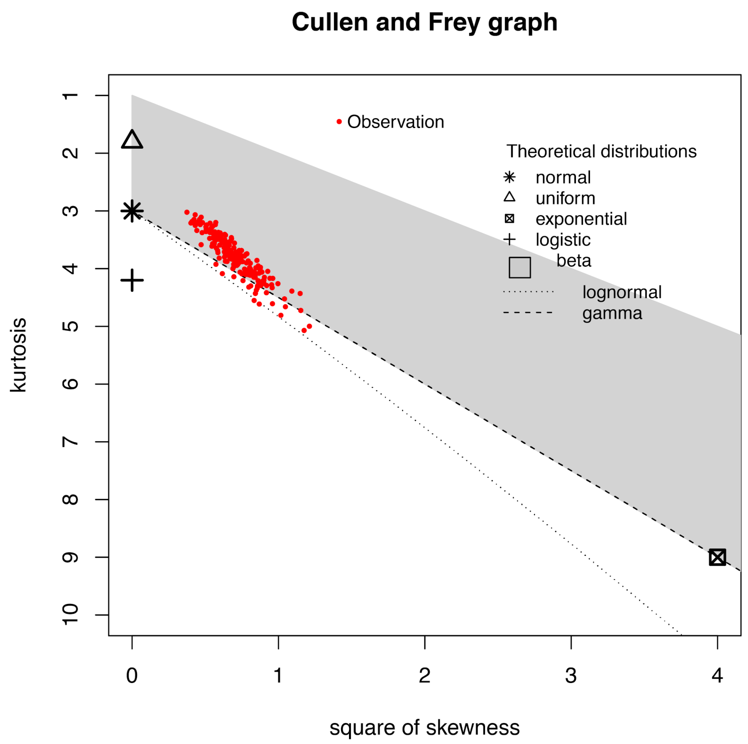


**Appendix Figure S13.**

Cullen and Frey graph for testing selected distributions of the Kolmogorov-Smirnov (KS) test statistics.

Each red dot represents distribution of KS test statistics between two NB random variables with distinct mean and dispersion parameters obtained from 1000 resamples. The mean and dispersion values used for generating the random variables were derived from mean-variance modelling of null-80 samples using DESeq2.
