# Supplementary material for: Improved detection of differentially represented DNA barcodes for high‐throughput clonal phenomics
Source: Mol Syst Biol. 2020 Mar 18;16(3):e9195. doi: 10.15252/msb.20199195 (PMC7080434; doi:10.15252/msb.20199195)
Supplement: Supplementary file 2 — Expanded View Figures PDF [file MSB-16-e9195-s002.pdf]

Expanded View Figures

Figure EV1. Clone size characteristics of the benchmark datasets.

A Cumulative distributions of clone sizes in OVCAR-5 null-660 sample (left) and Mia-Paca-2 null-40 sample (right).

B Barcode representation fold changes (log_2) for the null samples of the indicated sizes (number of cells subsampled from the AB mix) relative to the mean of two Null-660 replicas. Barcodes are ordered according to the size in the Null-660 subsamples. Pool A barcodes are sorted in the descending order, and Pool B barcodes are ordered in the ascending order. Boxes represent interquartile ranges (25 to 75 percentile) for each group of 53 observations. Whiskers indicate upper and lower quartiles. Central line corresponds to the median value.

C Same as Fig EV1B but for the perturbed subsamples. Dotted lines indicates the expected barcode fold changes calculated using formula: (cells from pool A/total number of cells)/0.5, for the Pool A barcodes, and formula: (cells from pool B/total number of cells)/0.5, for the Pool B barcodes. Data representation is the same as in (B).

© 2020 The Authors

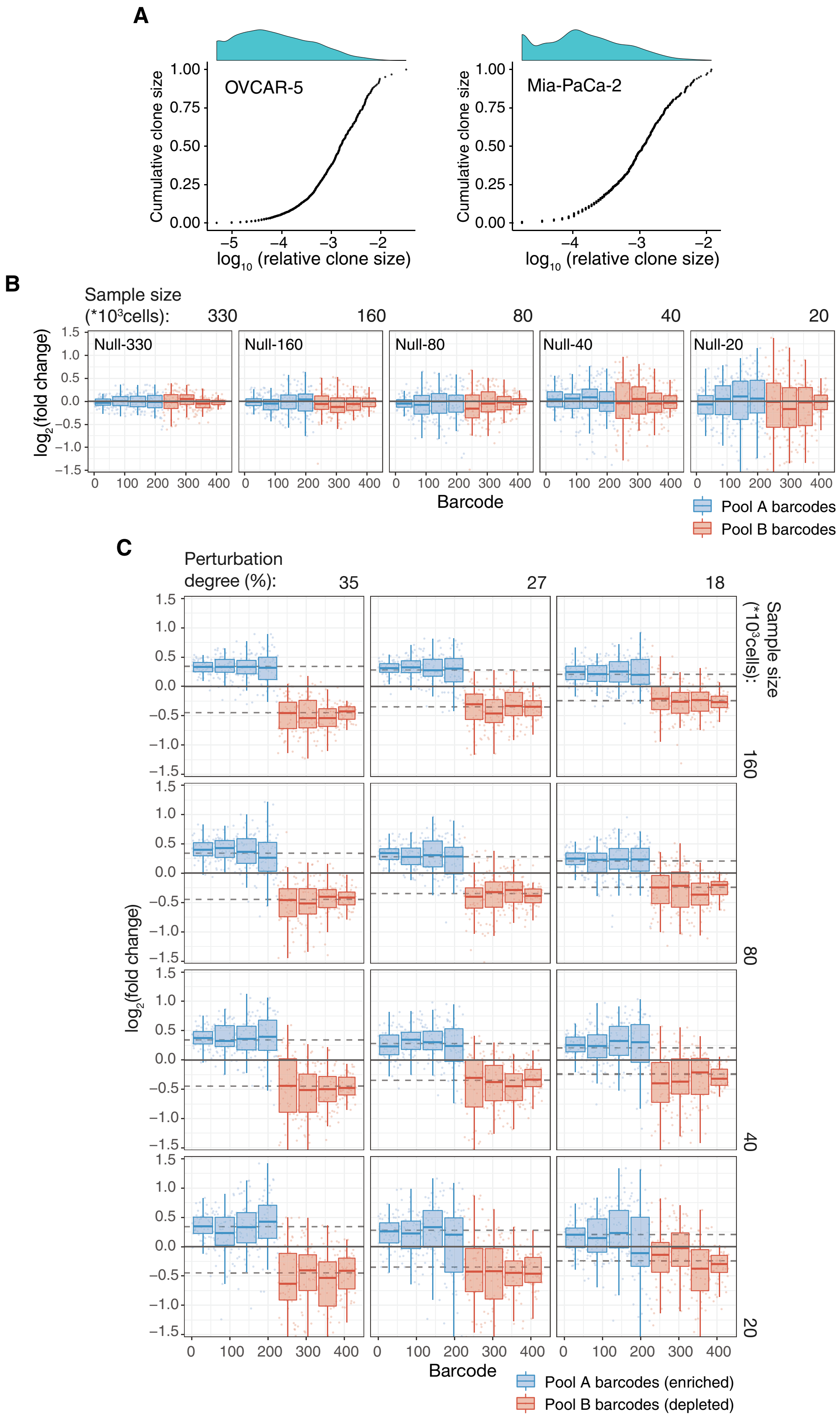

Figure EV1.

 © 2020 The Authors

Figure EV2. Sampling size affects statistical properties and accuracy of DRB calling..

A Mean–variance plots for the benchmark OVCAR5 null subsamples (replica#2) and perturbed subsamples (35% perturbation degree; replicas #1 and #2). Local variance was calculated by averaging a tagwise variance over the mean counts using a 20 read-count window. Mean counts were estimated using all the null or perturbed samples, respectively.

B Mean–variance plots for Mia-PaCa-2 null subsamples. Barcode read counts were median-normalized. Local variance was calculated by averaging a tagwise variance over the mean counts using a 20 read-count window.

C Scatter plots of median-normalized read counts of Mia-PaCa-2 null subsamples.

D Local negative binomial goodness of fit was estimated using chi-squared test or Cramer–von Mises test. Dispersion parameter of the negative binomial model was estimated locally over the window of 3 read counts using maximum-likelihood estimator. *P*-value of the chi-squared test statistics was estimated using fitdistrplus::gofstat() function. *P*-values of the Cramer–von Mises test were calculated by Monte Carlo bootstrap method as implemented in RVAideMemoire::cramer.test.

© 2020 The Authors

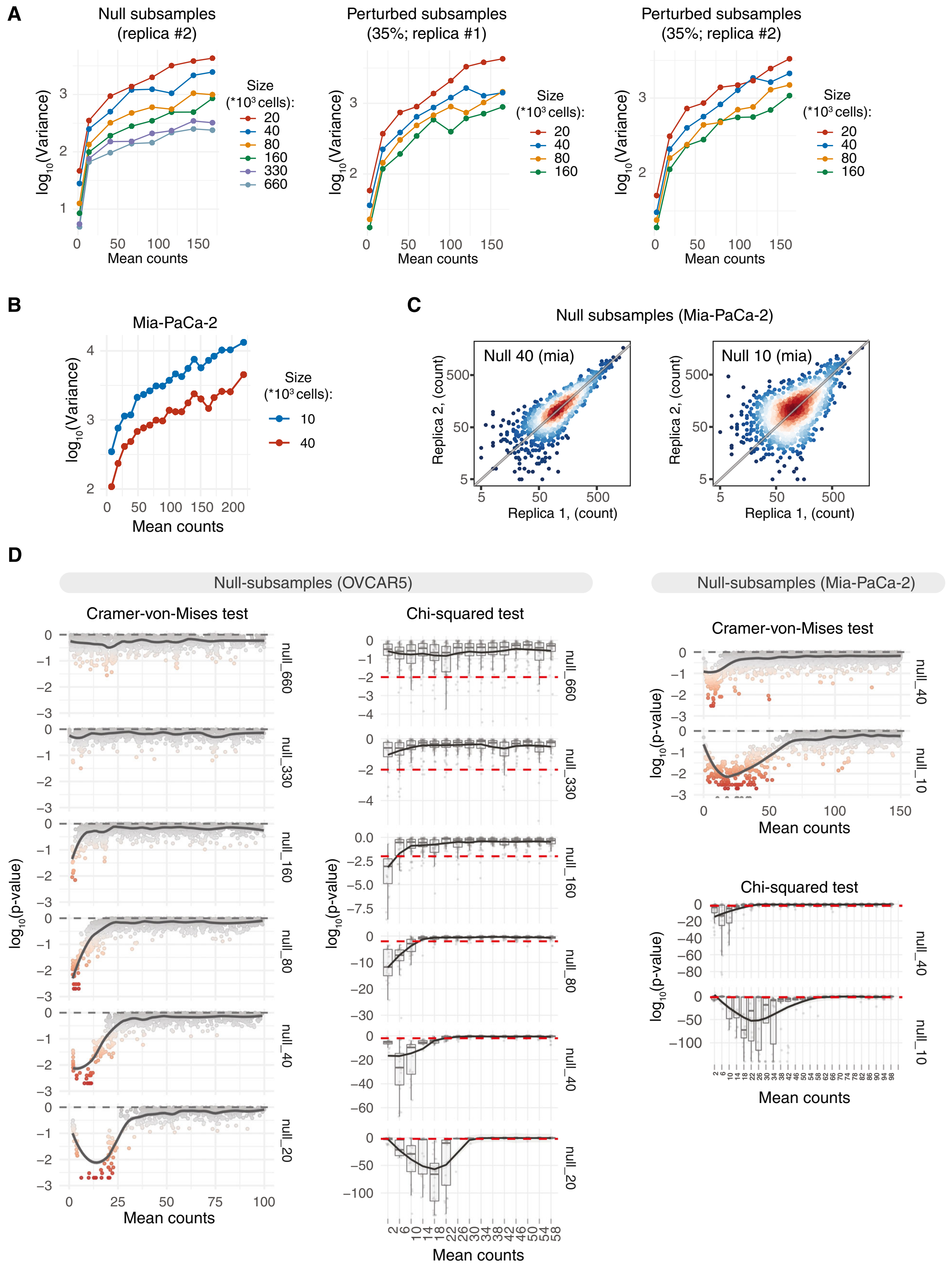

Figure EV2.

 © 2020 The Authors

Figure EV3. Comparison of the algorithm' performance on the perturbed subsamples.

A–C Circles left to the algorithms' names indicate the modified algorithms. Two replicas of the perturbed subsamples of indicated sample size (top), perturbation degree (right) and enriched barcode ratios of 0.05 (A), 0.15 (B) and 0.5 (C) were tested for DRBs against four control samples (two Null-660 samples and two Null-330 samples). Bars represent the average proportion of DRBs classified as enriched (fold change > 0) under the FDR threshold of 0.25, calculated over threefold bootstrap runs (10 resamples with replacement). Red bars indicate the average fraction of false positives (incorrectly assigned to the enriched group). Black lines indicate the "random" FDR—the average fraction of false discoveries observed when *P*-values were randomly permuted over the barcodes. White points indicate the nominal FDR threshold of 0.25. Dashed vertical lines indicate the total proportion of enriched barcodes. Error bars, SD.

© 2020 The Authors

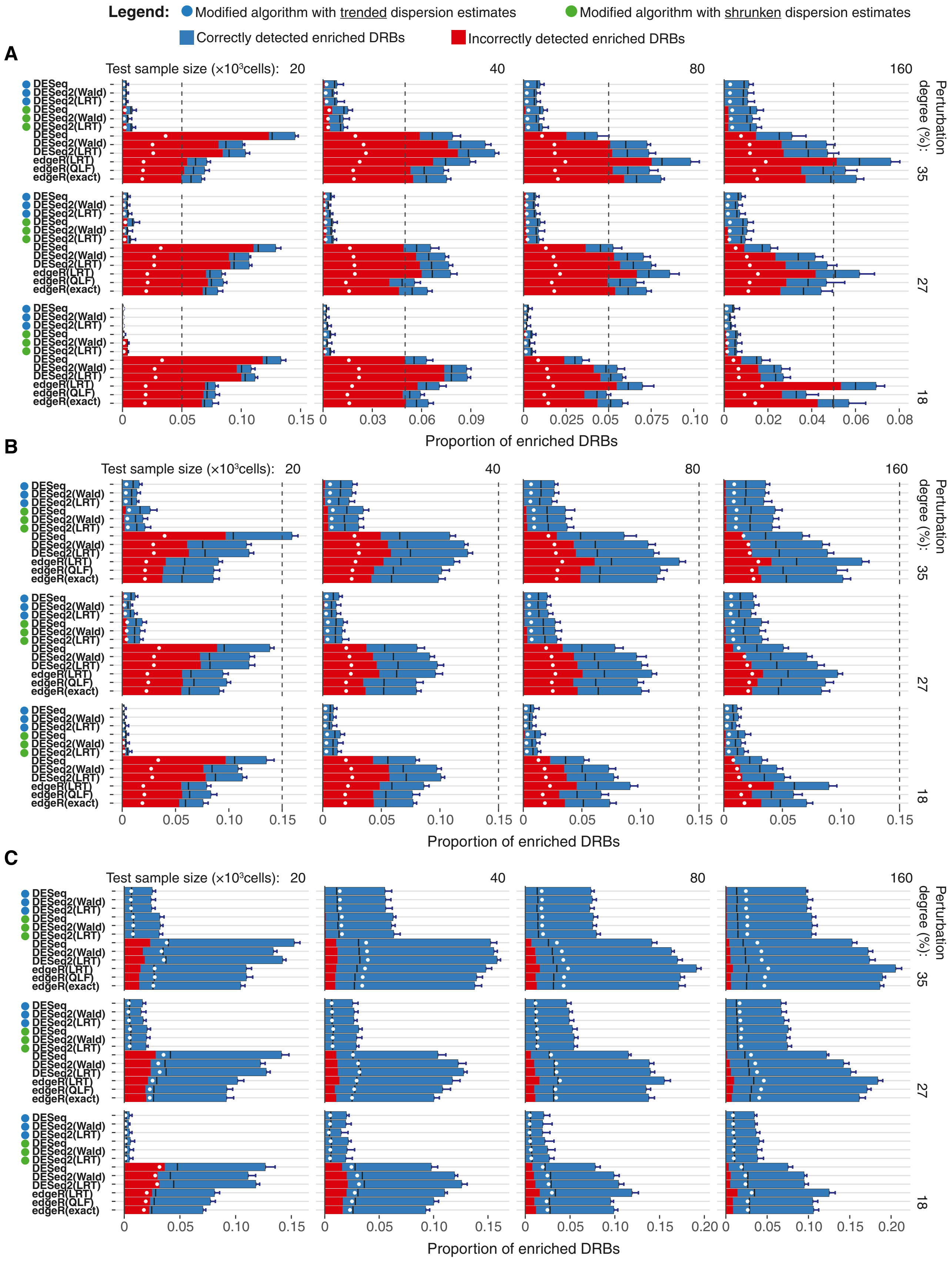

Figure EV3.

© 2020 The Authors

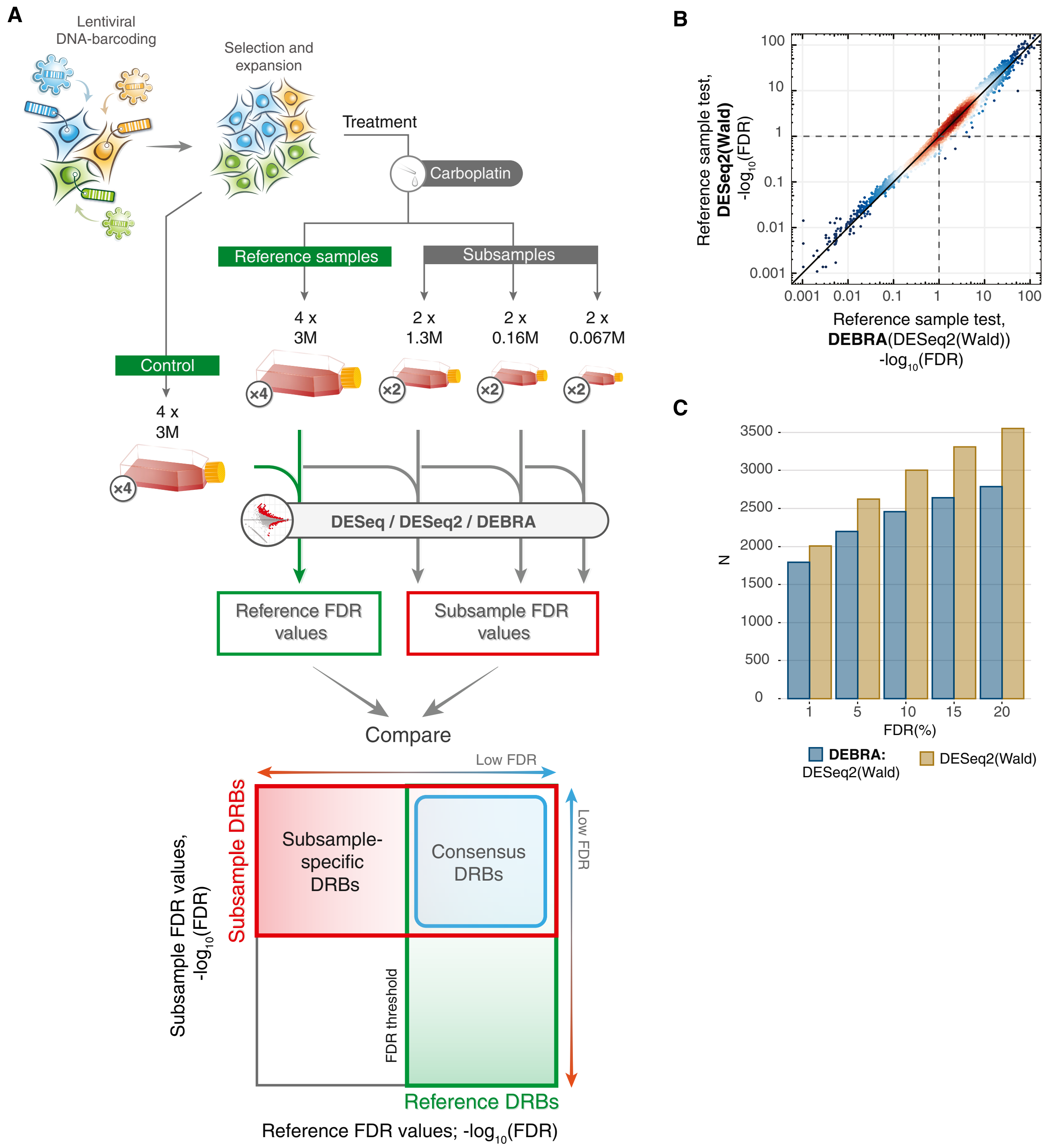

Figure EV4. Design of the carboplatin phenotyping experiments and the reproducibility of the DRB detection in the reference sample.

A Overview of the experimental design for the benchmark carboplatin phenotyping experiment. Cells were barcoded and expanded to achieve the average number of 1,000 cells per clone (see Materials and Methods). Next, the cells were divided into control samples (4 × 3 M), reference samples (4 × 3 M) and subsamples (2 × 1.33 M; 2 × 0.16 M and 2 × 0.067 M). Reference and subsamples were treated with carboplatin (IC50; 7 μM) for 4 days and then allowed to re-grow for 4 days. Barcode read counts from treated samples were tested against control samples with modified and non-modified algorithms. Obtained FDR values were then compared to detect the consensus DRBs (consistently detected in both subsamples and reference samples; outlined with blue square) and subsample-specific DRBs (detected only in the subsample but not in the reference sample). The number of replicas for each sample is indicated in circles next to the tube icon.

B Scatter plot of the FDR values detected with the DEBRA-modified DESeq2(Wald) versus original DESeq2(Wald) algorithms.

C The number of DRBs identified with different FDR thresholds using the DEBRA-modified DESeq2(Wald) or original DESeq2(Wald).

© 2020 The Authors

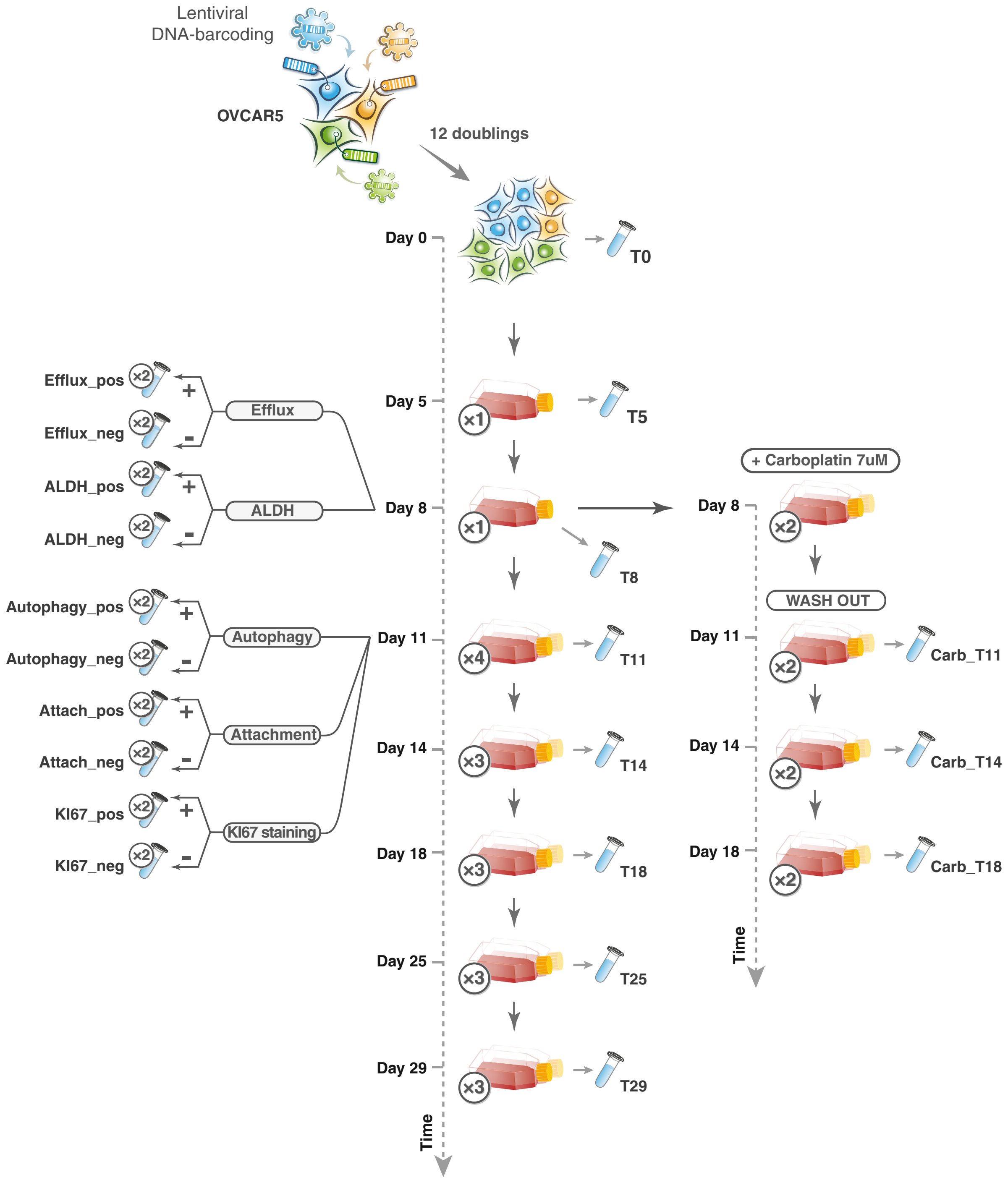

Figure EV5. A schematic representation of OVCAR5 clonal phenotypic profiling experiment.

In the proliferation assay, 3 million cells were plated in each passage, and at day 8, the population was split into 4 replicas. The tube image indicates that the sample was collected for sequencing. Sample name as used in the read count table (Dataset EV4) is marked next to the tube images. The number of replicas is indicated in the circles next to the flask/tube icons.

© 2020 The Authors

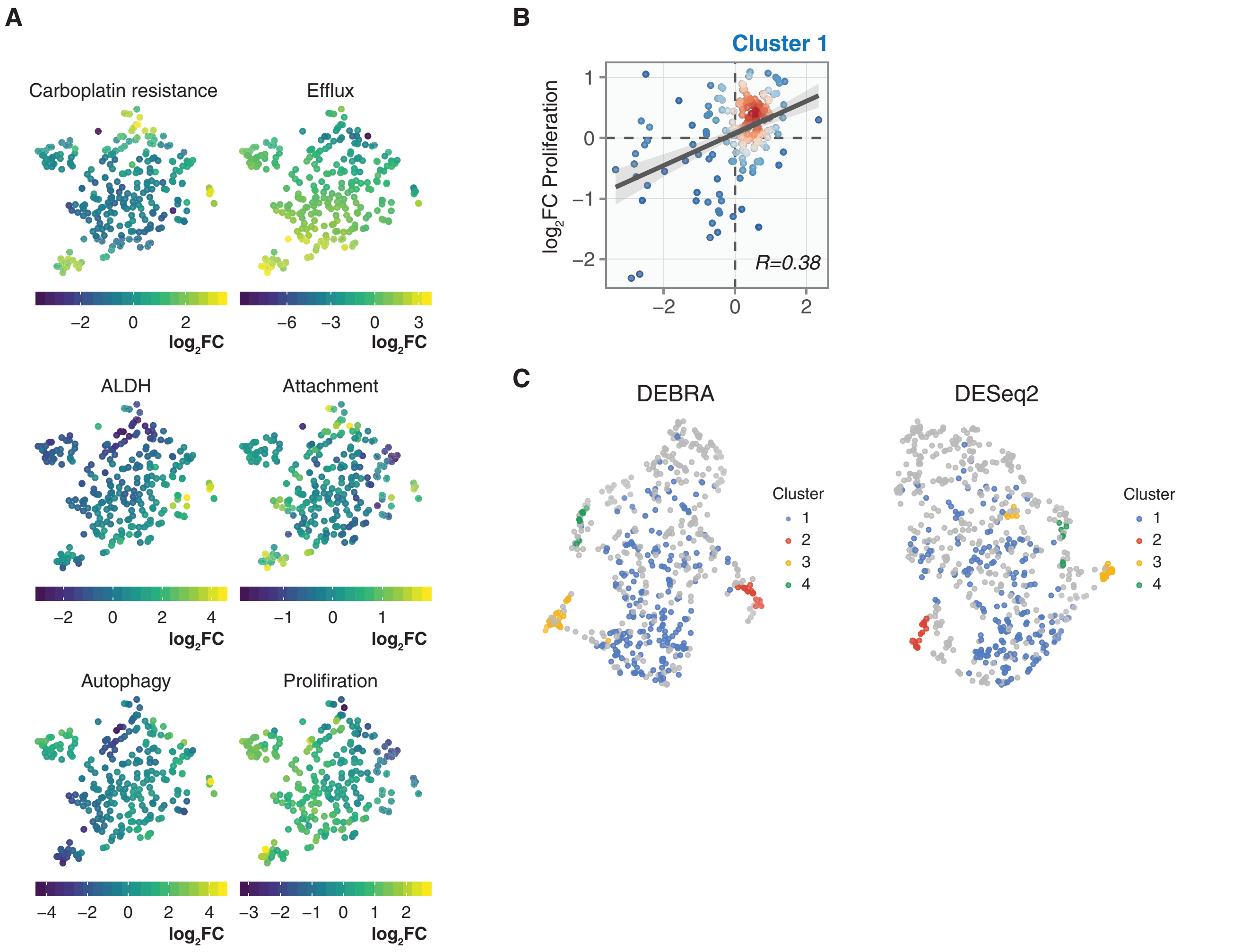

Figure EV6. Barcode fraction fold changes for OVCAR5 single-clone phenotypes.

A t-SNE projections of the OVCAR5 clonal phenotypic profiles. The clones are colour-coded according to the manifestation of the phenotype, calculated as log_2 ratio of barcode counts between (1) positively and negatively selected populations after ALDH, attachment, efflux capacity or autophagy assays; (2) treated and untreated samples (T14) for carboplatin treatment assay; or (3) day 8 and day 1 time points for proliferation assay.

B Scatter plot of barcode fraction fold changes in OVCAR5 cells in response to carboplatin treatment and after 7 days of growth assay for cluster 1 (Bulk; see Fig 5F).

C UMAP projections of the OVCAR5 clonal phenotypic profiles for clones selected according to the FDR level as determined by the DEBRA (left) or DESeq2 (right). The barcode was selected for the analysis if the minimum FDR value from the carboplatin, ALDH and efflux assays was less than 0.1. The clones are colour-coded according to clusters as defined at Fig 5F.

© 2020 The Authors
